# Supplementary material for: Predictive value of increased C-reactive protein levels in preterm infants on respiratory function at five to six years of age
Source: Commun Med (Lond). 2026 Apr 1;6:183. doi: 10.1038/s43856-026-01569-8 (PMC13046779; doi:10.1038/s43856-026-01569-8)
Supplement: Supplementary file 2 — Supplementary Material [file 43856_2026_1569_MOESM2_ESM.pdf]

Supplementary material

Supplementary Table 1: Cohort characteristics

Variable	No Elevation of CrP<br>n = 202	Single elevation of CrP > 10mg/l<br>n = 34	Recurrent elevation of CrP > 10 mg/l<br>n = 32	Total cohort<br>n = 268
Gestational age [weeks][#]	27.9 (26.1-29.7)	27.2 (25.3-29.1)	25.8 (24.5-27.4)	27.6 (25.7-29.4)
Birth weight [g][#]	980 (770-1249)	815 (730-1210)	723 (560-905)	948 (723-1219)
SGA (<10th percentile)	25 (12.4 [8.4-17.4])	8 (23.5 [11.8-39.5])	10 (31.3 [17.3-48.4])	43 (16.0 [12.0-20.8])
Female sex	94 (46.5 [39.7-53.4])	15 (44.1 [28.5-60.7])	12 (37.5 [22.4-54.8])	121 (45.1 [39.3-51.1])
Multipara	87 (43.1 [36.4-50.0])	16 (47.1 [31.1-63.5])	12 (37.5 [22.4-54.8])	115 (42.9 [37.1-48.9])
Antenatal administration of steroids	180 (89.1 [84.3-92.8])	33 (97.1 [87.1-99.7])	30 (93.8 [81.4-98.7])	243 (90.7 [86.8-93.7])
Surfactant administration	158 (78.2 [72.2-83.5])	30 (88.2 [74.4-95.9])	31 (96.9 [86.3-99.7])	219 (81.7 [76.8-86.0])
ICH	29 (14.4 [10.0-19.7])	4 (11.8 [4.1-25.6])	9 (28.1 [14.9-45.1])	42 (15.7 [11.7-20.4])

PVL	1 (0.5 [0.1-2.3])	1 (2.9 [0.3-12.9])	2 (6.3 [1.3-18.6])	4 (1.5 [0.5-3.5])
NEC requiring surgery	1 (0.5 [0.1-2.3])	1 (2.9 [0.3-12.9])	5 (15.6 [6.2-30.9])	7 (2.6 [1.2-5.1])
SIP requiring surgery	1 (0.5 [0.1-2.3])	0 (0)	4 (12.5 [4.4-27.0])	5 (1.9 [0.7-4.1])
invasive ventilation	99 (49.0 [42.2-55.9])	23 (67.6 [51.0-81.4])	31 (96.6 [86.3-99.7])	153 (57.1 [51.1-62.9])
Clinical signs of chorioamnionitis	47 (23.3 [17.8-29.4])	6 (21.6 [7.7-32.8])	8 (25.0 [12.6-41.7])	61 (22.8 [18.1-28.1])
Amount of CrP values > 10 mg/l	0	1 (1)	4 (3-9)	0 (0-1)
Frequency of CrP determinations	4 (3-6)	7 (5-9)	12 (8-14)	5 (3-8)

Supplementary table 1: Cohort characteristics stratified by amount of CrP levels > 10 mg/l, categorical variables are given as n(%) with corresponding 95% confidence interval (CI), continuous variables as median (IQR) if appropriate (#). Abbreviation: CrP: C-reactive protein, SGA: small for gestational age, ICH: intracerebral hemorrhage, PVL: periventricular leucomalacia, NEC: necrotizing enterocolitis, SIP: spontaneous intestinal perforation

Supplementary Table 2: Univariate analyses of outcome parameters and CrP levels

Variable	No Elevation of CrP (reference)<br><br>n = 202	Single elevation of CrP > 10mg/l<br><br>n = 34	Recurrent elevation of CrP > 10 mg/l<br>n = 32	p-value[1]
BPD	33 (16.8 [12.0-22.4])	10 (29.4 [16.2-45.9])	18 (56.3 [39.1-72.3])	0.080<br>< 0.001<br>0.027
Oxygen need at discharge	0 (0)	2 (5.9 [1.2-17.6])	4 (12.5 [4.4-27.0])	0.001<br>< 0.001<br>0.350
Non-invasive ventilation at discharge	1 (0.5 [0.1-3.0])	0	2 (6.3 [1.3-18.6])	0.680<br>0.007<br>0.139
Weight at 5YFU [kg]	18.6 (17.0-20.8)	18.8 (16.7-19.4)	16.7 (15.1-19.2)	0.860<br>0.002<br>0.027
IQ < 85	26 (13.8 [9.5-19.3])	4 (16.0 [5.7-33.7])	3 (11.1 [3.2-26.8])	0.769<br>0.699<br>0.606

GMFCS ≥ 1	30 (16.2 [11.4-22.0])	6 (18.8 [8.2-34.6])	10 (34.5 [19.3-52.6])	0.722<br>0.019<br>0.163
FEV_1 [litres]	0.98 (0.85-1.12)	0.93 (0.80-1.06)	0.77 (0.67-0.91)	0.411<br>< 0.001<br>0.009
FVC [l]	1.06 (0.92-1.23)	1.01 (0.83-1.15)	0.8 (0.66-0.94)	0.236<br>< 0.001<br>0.002
z-score (FEV_1) 50th percentile	-1.3 (-2.0-(-0.5))	-1.7 (-2.2 –(-0.7))	-2.5 (-3.0-(-1.5))	0.249<br>< 0.001<br>0.002
z-score (FVC) 50th percentile	-1.3 (-2.1-(-0.5))	-1.7 (-2.4 –(-0.6))	-2.7 (-3.3-(-1.9))	0.330<br>< 0.001<br>< 0.001
z-score (FEV_1) < 5th percentile	70 (34.7 [28.3-41.4])	21 (61.8 [45.0-76.6])	23 (71.9 [54.9-85.1])	< 0.001<br>< 0.001<br>0.384
z-score (FVC) < 5th percentile	74 (36.6 [30.2-43.4])	17 (50.0 [33.8-66.2])	26 (81.3 [65.4-91.8])	< 0.001<br>< 0.001<br>0.008
Distance run < 5th percentile	9 (7.7 [3.9-13.6])	2 (12.5 [2.7-34.4])	4 (22.2 [8.0-44.6])	0.513<br>0.021<br>0.458

**Distance run < 15th percentile**	25 (21.4 [14.7-29.4])	5 (31.3 [13.1-55.6])	7 (38.9 [19.4-61.7])	0.375<br>0.075<br>0.642

Supplementary table 2: Descriptive outcome of former preterm infants at the age of five to six years stratified by amount of CrP levels > 10 mg/l; categorical variables are given as n(%) with corresponding 95% confidence interval [95CI], continuous variables as median (IQR) if appropriate. [1]upper line: p-value of single CrP > 10 mg/l vs. no elevation of CrP, middle line: p-value of recurrent CrP > 10 mg/l vs. no elevation of CrP, lower line: p-value of recurrent CrP > 10 mg/l vs. single elevation of CrP. P-values derived from Pearson's Chi-square test or Mann-Whitney U-Test for continuous outcome. All tests were two-sided, and p-values < 0.05 were considered statistically significant. Abbreviation: 5YFU: five to six year follow-up examination, IQ: intelligence quotient, GMFCS: gross motor function scale, FEV_1: forced expiratory volume in one second

Supplementary Table 3a: Linear regression model for respiratory long-term outcome and single CrP elevations > 10 mg/l

Variables	z-score of FEV_1			
	B (SD)	Beta	T	p-value
GMFCS ≥ 1	-0.198 (0.227)	-0.069	-0.873	0.384
Weight at 5YFU [kg]	0.018 (0.021)	0.066	0.852	0.396
IQ	0.015 (0.006)	0.180	2.406	0.017
Duration of mechanical ventilation [days]	-0.204 (0.090)	-0.274	-2.270	0.025
Single CrP > 10 mg/l	-0.052 (0.248)	-0.016	-0.209	0.834

Supplementary table 3a: Linear regression model for respiratory long-term outcome. Model further adjusted for gestational age, birth weight, antenatal administration of steroids, IVH, surgical treatment for necrotizing enterocolitis, birth weight < 10th percentile, duration of mechanical ventilation, duration of oxygen therapy within the first 28 days, use of postnatal corticosteroids (dexamethasone and/or hydrocortisone), BPD and PVL (data not shown). R^2: 0.217; Durbin-Watson: 1.955; F=2.891 with $p < 0.001$; Abbreviation: B: unstandardized coefficients, Beta: standardized coefficients, IQ: intelligence quotient

Supplementary Table 3b: Linear regression model for respiratory long-term outcome

Variables	z-score of FVC			
	B (SD)	Beta	T	p-value
GMFCS ≥ 1	-0.470 (0.241)	-0.154	-1.951	0.053
Weight at 5YFU [kg]	0.034 (0.022)	0.119	1.546	0.124
IQ	0.015 (0.007)	0.116	2.229	0.027
Duration of mechanical ventilation [days]	-0.180 (0.095)	-0.227	-1.188	0.061
Single CrP > 10 mg/l	0.020 (0.263)	0.006	0.075	0.940

Supplementary table 3b: Linear regression model for respiratory long-term outcome. Model further adjusted for gestational age, birth weight, antenatal administration of steroids, IVH, surgical treatment for necrotizing enterocolitis, birth weight < 10th percentile, duration of mechanical ventilation, duration of oxygen therapy within the first 28 days, use of postnatal corticosteroids (dexamethasone and/or hydrocortisone), BPD and PVL (data not shown). R^2: 0.151; Durbin-Watson: 2.004; F=3.032 with $p < 0.001$; Abbreviation: B: unstandardized coefficients, Beta: standardized coefficients, IQ: intelligence quotient

Supplementary Table 4: Blood count grouped by single and recurrent CrP elevations

Variable	Single CrP elevation	Recurrent CrP elevations	p-value
Leukocytes	8043 (4290-12335)	17770 (11080-20195)	0.006
Segmented neutrophils	3468 (1838-4800)	6166 (5504-10289)	0.025
Banded neutrophils	210 (114-301)	779 (335-1084)	0.006
Eosinophils	290 (218-442)	560 (330-908)	0.080
Lymphocytes	3632 (1722-4509)	4171 (3582-5441)	0.295
Platelets	164 (141-210)	287 (195-383)	0.095

Supplementary table 4: White blood count measurements differentiated by single and recurrent CrP elevations > 10 mg/l. Values given as median (IQR). P-values derived from Mann-Whitney U-Test. All tests were two-sided, and p-values < 0.05 were considered statistically significant.

Supplementary Table 5a: Linear regression model for respiratory long-term outcome and median CrP levels

Variables	z-score of FEV_1			
	B (SD)	Beta	T	p-value
GMFCS ≥ 1	-0.133 (0.202)	-0.047	-0.655	0.513
Weight at 5YFU [kg]	0.013 (0.019)	0.049	0.684	0.495
IQ	0.012 (0.006)	0.138	2.030	0.044
Duration of mechanical ventilation [days]	-0.240 (0.084)	-0.317	-2.862	0.005
Median CrP levels	-0.009 (0.019)	-0.038	-0.497	0.620

Supplementary table 5a: Linear regression model for respiratory long-term outcome. Model further adjusted for gestational age, birth weight, antenatal administration of steroids, IVH, surgical treatment for necrotizing enterocolitis, birth weight < 10th percentile, duration of mechanical ventilation, duration of oxygen therapy within the first 28 days, use of postnatal corticosteroids (dexamethasone and/or hydrocortisone), BPD and PVL (data not shown). R^2: 0.246; Durbin-Watson: 1.717; F=3.947 with $p < 0.001$; Abbreviation: B: unstandardized coefficients, Beta: standardized coefficients, IQ: intelligence quotient

Supplementary Table 5b: Linear regression model for respiratory long-term outcome

Variables	z-score of FVC			
	B (SD)	Beta	T	p-value
GMFCS ≥ 1	-0.567 (0.229)	-0.173	-2.471	0.014
Weight at 5YFU [kg]	0.048 (0.022)	0.154	2.165	0.032
IQ	0.012 (0.007)	0.113	1.690	0.093
Duration of mechanical ventilation [days]	-0.237 (0.095)	-0.271	-2.486	0.014
Median CrP levels	-0.018 (0.021)	-0.066	-0.869	0.386

Supplementary table 5b: Linear regression model for respiratory long-term outcome. Model further adjusted for gestational age, birth weight, antenatal administration of steroids, IVH, surgical treatment for necrotizing enterocolitis, birth weight < 10th percentile, duration of mechanical ventilation, duration of oxygen therapy within the first 28 days, use of postnatal corticosteroids (dexamethasone and/or hydrocortisone), BPD and PVL (data not shown). R^2: 0.273; Durbin-Watson: 1.903; F=4.527 with p < 0.001; Abbreviation: B: unstandardized coefficients, Beta: standardized coefficients, IQ: intelligence quotient

Supplementary Table 6: Characterization of infants with single and recurrent CrP elevations but no diagnosis of BPD

Variable	No Elevation of CrP (reference)<br><br>n = 227	Single elevation of CrP > 10mg/l<br><br>n = 34	Recurrent elevation of CrP > 10 mg/l<br>n = 23	p-value*
Gestational age [weeks]#	28.4 (26.9-29.7)	28.4 (25.4-29.3)	25.9 (24.4-27.3)	0.037
Birth weight [g]#	1025 (830-1270)	923 (730-1293)	815 (602-970)	0.118
SGA (<10th percentile)	18 (10.6 [6.6-15.9])	6 (23.1 [10.3-41.5])	4 (21.1 [7.6-42.6])	0.064
Female sex	87 (51.2 [43.7-58.6])	12 (46.2 [28.2-64.9])	6 (31.6 [14.4-53.9])	0.328
Multipara	72 (42.4 [35.1-49.9])	12 (46.2 [28.2-64.9])	6 (31.6 [14.4-53.9])	0.578
Antenatal administration of steroids	151 (88.8 [83.4-92.9])	25 (96.2 [83.4-99.6])	17 (89.5 [70.3-97.7])	0.634
Surfactant administration	127 (74.7 [67.8-80.8])	22 (84.6 [67.5-94.6])	18 (94.7 [77.9-99.4])	0.021
ICH	21 (12.4 [8.1-17.9])	1 (3.8 [0.4-16.6])	3 (15.8 [4.7-36.4])	0.852
PVL	1 (0.6 [0.1-2.7])	0	1 (5.3 [0.6-22.1])	0.074

NEC requiring surgery	1 (0.6 [0.1-2.7])	0	4 (21.1 [7.6-42.6])	< 0.001
SIP requiring surgery	1 (0.6 [0.1-2.7])	0 (2.9 [0.3-12.9])	1 (5.3 [0.6-22.1])	0.022
invasive ventilation	69 (40.6 [33.4-48.1])	15 (57.7 [38.7-75.0])	18 (94.7 [77.9-99.4])	< 0.001
Clinical signs of chorioamnionitis	38 (22.4 [16.6-29.0])	4 (15.4 [5.4-32.5])	5 (26.3 [10.8-48.4])	0.921
Oxygen need at discharge	0	0	1 (5.3 [0.6-22.1])	0.882
Non-invasive ventilation at discharge	1 (0.6 [0.1-2.7])	0	1 (5.3 [0.6-22.1])	0.247
Weight at 5YFU# [kg]	18.9 (17.1-20.9)	18.8 (16.6-19.8)	17.3 (13.8-19.7)	0.306
IQ < 85	18 (11.3 [7.1-16.8])	3 (15.0 [4.4-34.9])	3 (18.8 [5.6-42.1])	0.173
GMFCS ≥ 1	18 (11.5 [7.2-17.2])	4 (16.7 [5.9-34.9])	3 (18.8 [5.6-42.1])	0.024
FEV_1# [litres]	1.01 (0.86-1.12)	0.96 (0.80-1.06)	0.82 (0.62-0.95)	0.077
FVC# [l]	1.09 (0.93-1.26)	1.03 (0.83-1.18)	0.83 (0.62-0.97)	0.014
z-score (FEV_1) < 5th percentile	53 (31.2 [24.6-38.4])	14 (53.8 [35.1-71.8])	12 (63.2 [40.9-81.8])	0.005

**z-score (FVC) < 5th percentile**	55 (32.4 [25.7-39.6])	12 (46.2 [28.2-64.9])	15 (78.9 [57.4-92.4])	< 0.001

*Supplementary table 6: Baseline data and descriptive outcome of former preterm infants at the age of five to six years stratified by amount of CrP levels > 10 mg/l, categorical variables are given as n(%) with corresponding 95% confidence interval [95CI], continuous variables as median (IQR) if appropriate (#). P-values derived from Pearson's Chi-square test or Mann-Whitney U-Test for continuous outcome. All tests were two-sided, and p-values < 0.05 were considered statistically significant.. * p-values given for single CrP > 10 mg/l vs. recurrent CrP > 10 mg/l. Abbreviation: 5YFU: five to six year follow-up examination, IQ: intelligence quotient, GMFCS: gross motor function scale, FEV_1: forced expiratory volume in one second*

Supplementary Table 7: Respiratory symptoms within 12 months before 5-6 year follow-up examination

Variable	No Elevation of CrP (reference)<br><br>n = 202	Single elevation of CrP > 10mg/l<br><br>n = 34	Recurrent elevation of CrP > 10 mg/l<br>n = 32	p-value*
Asthma or obstructive bronchitis during the past 12 months before 5YFU	67 (30.6 [24.8-36.9])	12 (36.4 [21.6-53.4])	9 (31.0 [16.6-49.0])	0.658

*Supplementary table 7: Data from parental questionnaire of former preterm infants at the age of five to six years stratified by amount of CrP levels > 10 mg/l, parents were asked at the 5-6 year follow-up visit; categorical variables are given as n(%) with corresponding 95% confidence interval [95CI], continuous variables as median (IQR) if appropriate (#). P-values derived from Pearson's Chi-square test or Mann-Whitney U-Test for continuous outcome. * p-values given for single CrP > 10 mg/l vs. recurrent CrP > 10 mg/l. Abbreviation: 5YFU: 5-6 year follow-up*

Supplementary figure 1: Frequency of CrP determinations

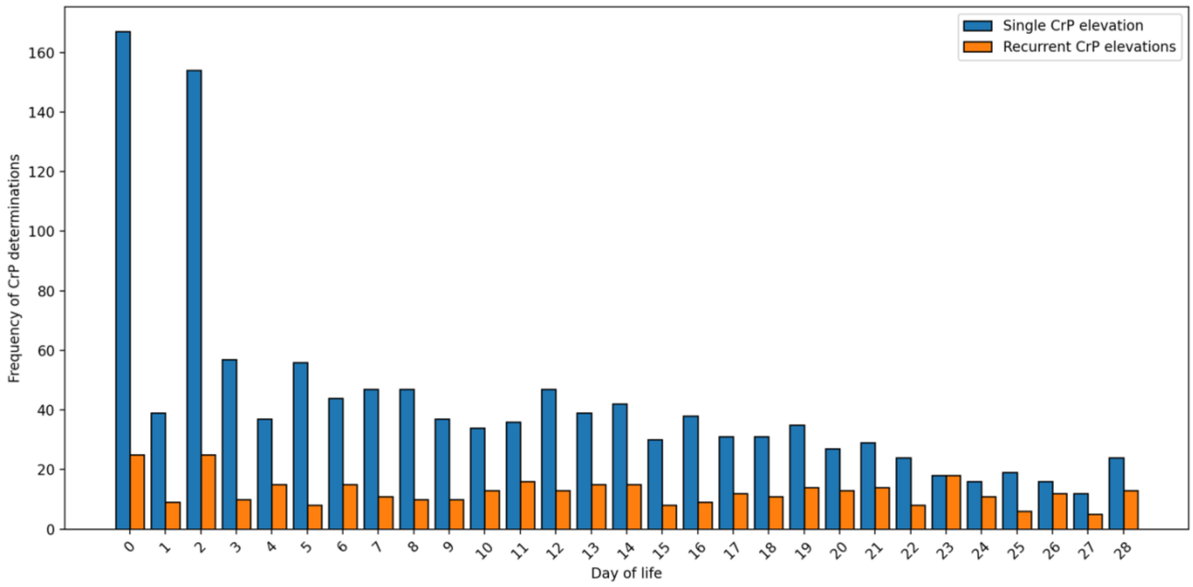

Supplementary figure 1: Frequency of CrP determinations within the first 28 days of life by day of life and grouped by recurrent elevations > 10 mg/l against all other infants. Absolute number of determinations in our cohort is presented.

Supplementary figure 2: CrP course over the first 28 days of life

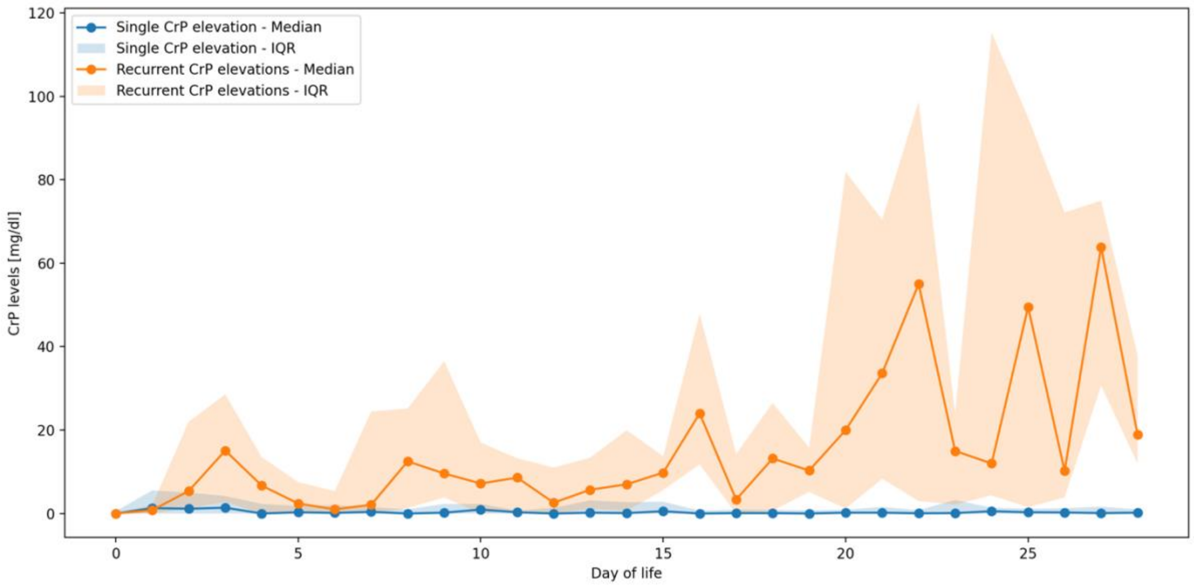

Supplementary figure 2: Course of median CrP levels with corresponding IQR over the first 28 days of life grouped by single and recurrent elevations of CrP.

Supplementary figure 3: CrP course over the first 28 days of life

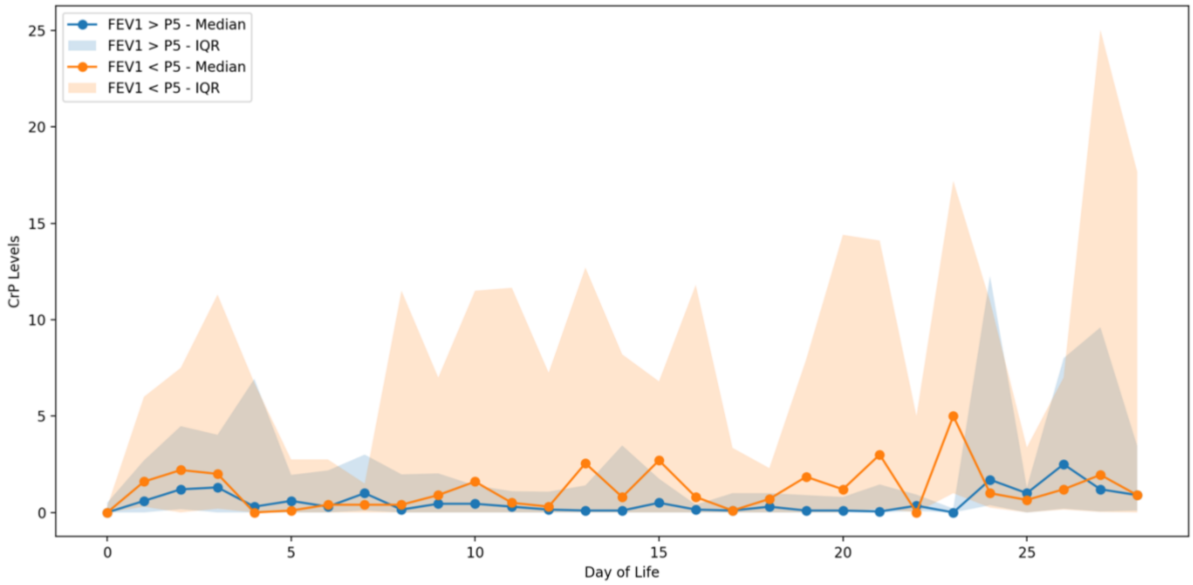

Supplementary figure 3: Course of median CrP levels with corresponding IQR over the first 28 days of life grouped by FEV1 < 5th percentile and FEV1 > 5th percentile.

Supplementary figure 4: CrP course over the first 28 days of life

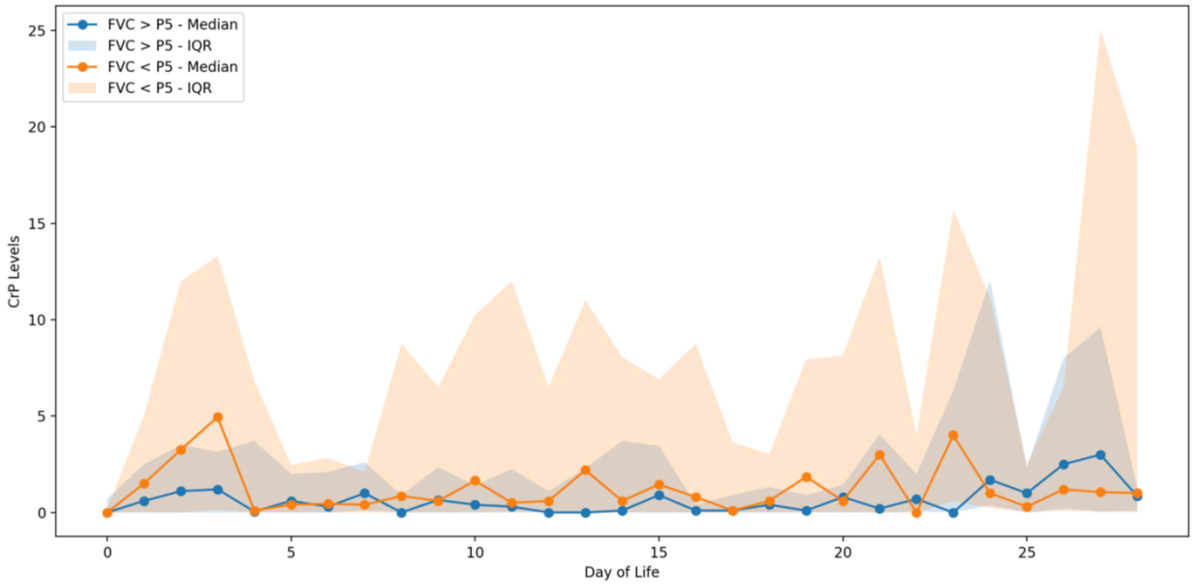

Supplementary figure 4: Course of median CrP levels with corresponding IQR over the first 28 days of life grouped by FVC < 5th percentile and FVC > 5th percentile.
